# Near-field microwave sensing technology enhanced with machine learning for the non-destructive evaluation of packaged food and beverage products

**DOI:** 10.1038/s41598-024-62287-6

**Published:** 2024-06-11

**Authors:** Ali Darwish, Marco Ricci, Jorge A. Tobon Vasquez, Claire Migliaccio, Francesca Vipiana

**Affiliations:** 1 Department of Electronics and Telecommunications, Politecnico di Torino, 10129 Turin, Italy; 2 LEAT, Université Côte d’Azur, 06903 Sophia Antipolis, France

**Keywords:** Microwave sensing, Non-destructive technique, Electromagnetic modeling, Antenna, Machine learning, Near-field sensing, Electrical and electronic engineering, Physics

## Abstract

In the food industry, the increasing use of automatic processes in the production line is contributing to the higher probability of finding contaminants inside food packages. Detecting these contaminants before sending the products to market has become a critical necessity. This paper presents a pioneering real-time system for detecting contaminants within food and beverage products by integrating microwave (MW) sensing technology with machine learning (ML) tools. Considering the prevalence of water and oil as primary components in many food and beverage items, the proposed technique is applied to both media. The approach involves a thorough examination of the MW sensing system, from selecting appropriate frequency bands to characterizing the antenna in its near-field region. The process culminates in the collection of scattering parameters to create the datasets, followed by classification using the Support Vector Machine (SVM) learning algorithm. Binary and multiclass classifications are performed on two types of datasets, including those with complex numbers and amplitude data only. High accuracy is achieved for both water-based and oil-based products.

## Introduction

The production process within food industry has been significantly influenced by the rapid advancement of technology, particularly in the automation of various production stages. The packaging phase is deemed to be one of the crucial stages in the production chain, primarily due to the heightened risk of contaminants finding their way into food packages produced using materials like plastic, glass, and nylon^1^. In the context of food production’s unyielding commitment to safety, a significant challenge emerges in identifying physical contaminants in packaged food throughout the production chain. Companies that are operating in this domain have, therefore, intensified their efforts to implement rigorous safety procedures at the production level to ensure the quality of their products before sending them to the markets. The food industry requires in-line inspections, meaning that every product carried by the conveyor belt has to be checked in a non-destructive way. To attain this objective, many technologies and methodologies have been explored and employed. The X-ray inspection is among the most widely used technologies to address this issue. However, it falls short in detecting low-density intrusions^2^ and demands safety precautions to protect operators and food materials from the hazards of ionizing radiation. Also, the metal detector technology is used in the industry for this purpose^3^. However, it is limited to metal contaminants and is unable to detect non-metallic materials like glass or plastic, which are the primary substances that food comes into contact with during the production process. Alternative techniques such as near-infrared imaging (NIR)^4^ and terahertz imaging^5^ have been investigated for detecting contaminants within food packages. However, both techniques have limitations in terms of penetration depth, particularly when dealing with dissipative mediums^6^. Despite the various technologies and methods mentioned above, there is still a notable rise in the number of recalled food and beverage products, primarily attributed to physical contamination^7,8^. Given this significant increase, it is imperative for food industry companies to actively pursue and integrate new techniques to solve this issue.

Employing microwave (MW) sensing technology presents itself as a potentially effective and powerful alternative non-destructive technique. This approach has the potential to address the limitations of previously mentioned techniques, and it stands out as a favorable option due to its good penetration depth, low cost, and simplified system design compared to the other adopted technologies. MW sensing systems use low-power MW radiations to extract information about the dielectric properties of materials by emitting signals and analyzing the reflections and scattering of those signals. The real-time detection and the quality control goals necessitate an innovative system that is capable of seamlessly integrating with the conveyor belt in a production line chain in the food industry. Implementing MW sensing technology involves signals collected by antennas surrounding the test samples across various frequency points. In this context, the movement of the sample on the conveyor belt is advantageous because it allows collecting data from different angles and positions, enabling a comprehensive examination of the tested samples. On the other hand, employing the conveyor belt can pose challenges as it necessitates exceptionally rapid measurements and swift data processing. Applying MW sensing poses several challenges, making it a complex application in terms of antenna coupling effects, non-uniformity of the fields, and calibration challenges. The novel detection principle employed by this technology relies on the dielectric contrast between the background (the food medium) and the contaminant. The varying permittivities result in a modification of the MW scattering characteristics, which if accurately captured, acknowledges the presence of a foreign body inside food packages in a non-invasive way. Moreover, a MW sensing system has to be designed to operate in the near-field region of the antennas^9^.

Combining MW sensing with ML classification algorithms constitutes a potent and efficient approach in this domain. This integration has found applications in diverse fields including, remote sensing^10^, ground-penetrating radar (GPR)^11^, detection of water pollutants^12,13^, material identification^14^, and medical activities^15–19^. In recent years, there has been an exploration of applying MW sensing technology enhanced with ML, in the food industry^20^. In the literature^21^, researchers have used millimeter wave imaging in conjunction with support vector machine (SVM) algorithm to identify defects within apples and peaches. Additionally, MW scattering parameters have been employed alongside neural network classifiers in^22^ to differentiate between healthy and spoiled walnuts.

In previous studies^23–25^, the authors have developed and used a MW sensing system augmented with ML binary classifications to assess hazelnut-cocoa cream jars, which share dielectric properties similar to those of oil-based products. In this paper, the focus is instead on expanding the investigated range of food product ingredients, and as water and oil are the main two ingredients of various types of products in the food industry, we present a methodology for testing food and beverage items that are high in water content, which is a lossy medium, as well as those that are mainly made up of oil, which is a low-loss medium. Developing a system capable of encompassing measurements across a spectrum ranging from low-loss to lossy media with diverse permittivity ranges, and possessing the ability to detect physical contaminants of a few millimeters in size, presents a significant challenge, that, as the same time, has a great potential impact on the food industry. To align with our new objectives, we start from the system presented in^24,25^ by replacing the antenna array elements with antennas capable of effectively operating across a broadband frequency range. This wide-band frequency range enables coverage of various material media, including oil and water. Throughout this paper, we explain the different steps for designing and using a system that can be suitable for industrial non-invasive evaluation of packaged food products. The paper begins by providing a comprehensive overview of the new MW sensing system, followed by a detailed characterization of the employed antenna within its near-field range. This work establishes a measurement scheme for binary and multiclass classifications, capable of discerning between various contaminant types, and it concludes with the classification results using the SVM algorithm after having discussed the most relevant training datasets for oil-based and water-based products.

## Microwave sensing in-line inspection scanner

**Figure 1:**
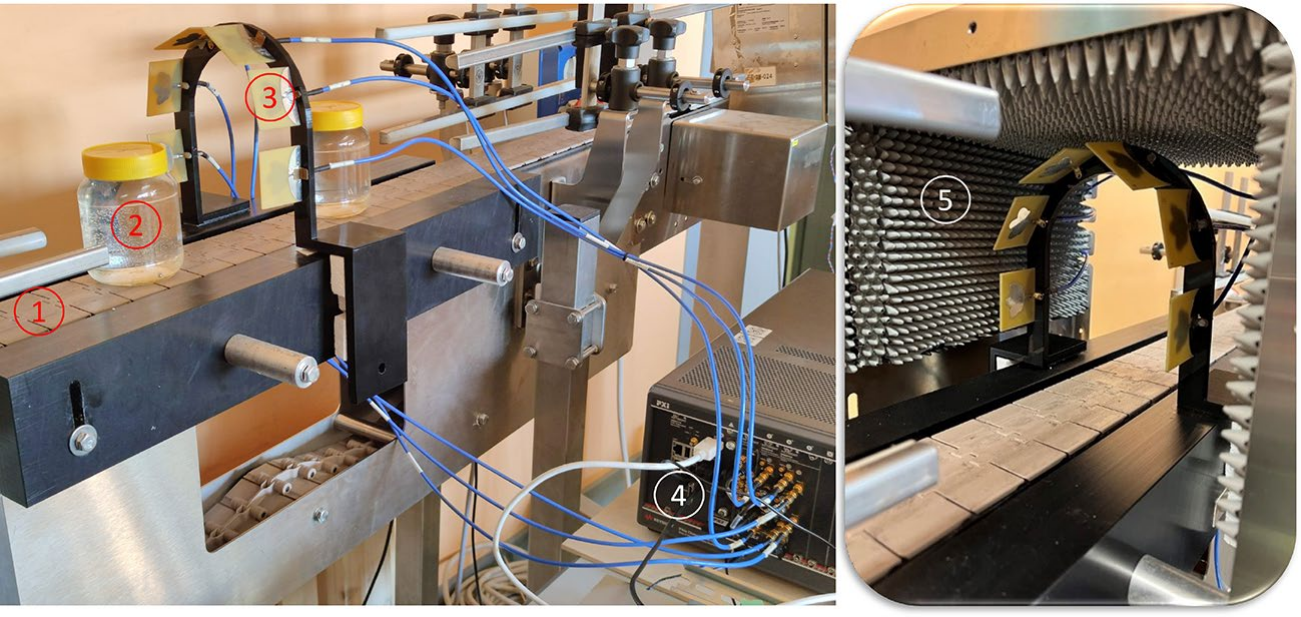
The complete measurement system comprises: (1) the conveyor belt, (2) the jar under test, (3) the antennas, (4) the vector network analyzer (VNA), (5) the shielding box.

The MW sensing system, illustrated in Fig. 1, includes six antennas mounted on an arch-shaped support that enables the sample to pass through without any interruption or delay. The antennas are connected to a six-port vector network analyzer (VNA)^26^ that captures the transmitted signal from the radiating antenna in complex numbers format at the operating frequency band. The antenna array setup is enclosed within a shielding box made up externally of metal to mitigate interference originating in the industrial environment, and internally of a layer of MW absorber that limits MW signal reflections. The speed of the conveyor belt in the food industry is around 20 meters per minute (that corresponds to $33\,\text {cm/s}$): this speed must be compatible with the measurement and data processing time. Moreover, initiating the measurement process as the jar approaches, the antenna array involves employing a photocell to trigger the VNA. The system counts with the photocell, and the measurement is triggered when the jar is in the desired position (through an automatic adapted delay). Variations on the speed of the belt are already considered by the control of the encoder. The measurement system is adapted to suit the measurement of lossy and low-loss mediums by using a custom-mode wide-band PCB-printed monopole antenna. In the following sections, we provide a detailed analysis of the frequency bandwidth selection, numerical investigation, and evaluation of the antenna in its near-field region.

### Frequency selection

The selection of the operating frequency bandwidth is a balance between the penetration depth of the electromagnetic (EM) waves, the dimensions of the food jar, and the desired resolution to be able to detect intrusions such as PolyTetraFluoroEthylene (PTFE, that is a kind of plastic common in packaging) and soda-lime glass (SLG) of millimeter size (detailed in Table 1).

**Table 1:** The range of the relative permittivity for the contaminants, used in the measurements, in the chosen frequency ranges.

Contaminants	Relative permittivity range ($\epsilon _r$)
PTFE	2–2.1
SLG	4.7–10

**Figure 2:**
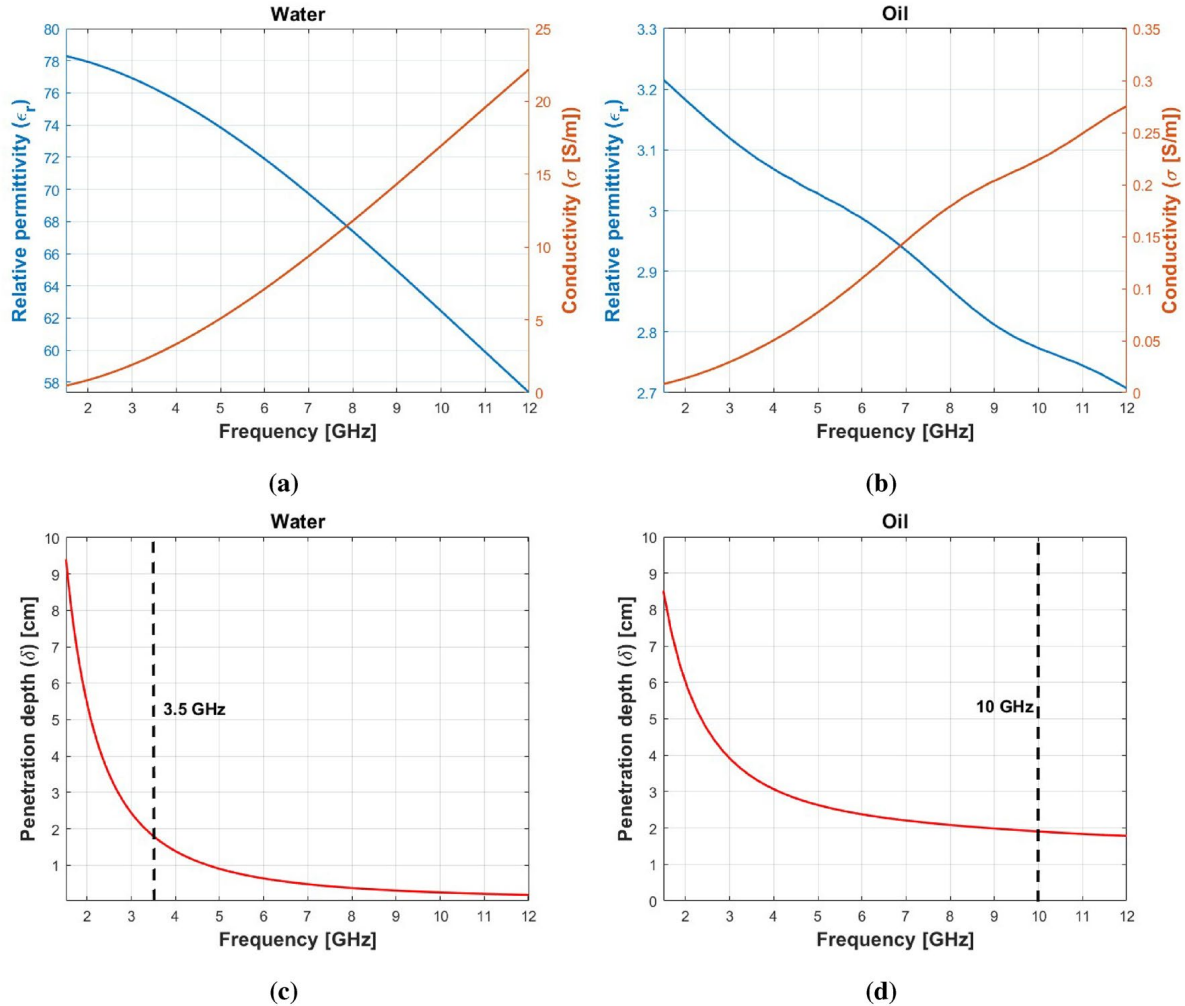
The EM characteristics of water and oil. (**a**) Relative permittivity and conductivity of water. (**b**) Relative permittivity and conductivity of oil. (**c**) Penetration depth within water. (**d**) Penetration depth within oil. The dotted lines within (**c,d**) denote the upper limit for the frequency range selected for carrying out our measurements on the samples.

Different frequencies may be appropriate for detecting different types of contaminants within food and beverage products. Shorter wavelength waves are more accurate in detecting smaller objects, because they have a higher spatial resolution that is inversely proportional to the wavelength, whereas longer wavelengths are more effective in lossy media due to their higher penetration capability. The dielectric property of the materials plays an important role in allowing these waves to go through these media or not. This property is frequency-dependent, which makes the selection of the frequency ranges for conducting the measurements very important. Hence, it is important to evaluate the dielectric characteristics of the two considered food materials, oil and water, along with the possible contaminants. For the measurements of the dielectric properties of oil and water, we use a 2-ports P9375A Keysight VNA^27^ along with 85070D Keysight dielectric probe together with the keysight software suite^28^. The results are shown in Fig. 2a, b. The relative permittivity of water significantly exceeds that of oil, resulting in a more pronounced dielectric contrast with respect to the contaminant materials shown in Table 1 compared to the contrasts between the permittivity of oil and the contaminants. This heightened contrast facilitates the detection of intrusions in water as opposed to oil. On the other hand, the conductivity values are notably higher in the case of water, indicating a reduced penetration depth within water compared to oil. High-loss materials present challenges for wave penetration. However, a good selection of the frequency operating bandwidth can solve this problem. The penetration depth, $\delta$, is reported in Fig. 2c, d and it is evaluated as follows:

1 $$\begin{aligned} \hspace{45mm} k = k_0\sqrt{\varepsilon _r - j\frac{\sigma }{\omega \varepsilon _0}} = \beta - j\alpha ,\quad \delta = \frac{1}{\alpha }, \end{aligned}$$

in which $k$ is the propagation constant, $k_0=\omega \sqrt{\varepsilon _0\mu _0}$ is the free space propagation constant, ${\omega }$ is the angular frequency, ${\varepsilon _0}$ and ${\mu _0}$ are the free space permittivity and permeability respectively, ${\varepsilon _r}$ and ${\sigma }$ are the medium relative permittivity and conductivity respectively. The penetration depth plots for water and oil indicate that is essential to fix an upper limit to avoid the very low penetration depth of the propagating waves inside the medium, particularly with water. Taking into account the considered diameter of the jar under analysis, which is around $8\,\text {cm}$, the dotted lines in Fig. 2c, d represent the upper limit for selecting frequency bands for both materials. On the other hand, the lower limit of the frequency bands should consider the necessary resolution for detecting millimeter-sized intrusions, considering the different dielectric permittivities of oil and water. Taking all these parameters into consideration, we set the operating frequency bandwidth from $6\,\text {GHz}$ to $10\,\text {GHz}$ for the oil-based food jars, and from $1.5\,\text {GHz}$ to $3.5\,\text {GHz}$ for the water-based food products.

Considering the minimum size of the contaminants (that is equal to 4 mm), we choose this dimension because it corresponds to the minimum size contaminants used to test detection systems in food industries, and the wavelength of the EM waves being used, the number of antennas required in MW sensing application is determined. In general, a higher number of antennas can lead to better sensing performance, as it provides more information about the object being radiated. However, a higher number of antennas increases the system complexity and cost. Therefore, it is important to find a trade-off between the number of antennas, sensing performance, and implementing considerations such as system size and cost. In the references^29,30^, it is estimated that the optimal configuration for this system involves the use of six antennas arranged in an arch shape. This arrangement has been proposed considering also the presence of the conveyor belt, the supporting structure for the belt and the dimensions of the food jar samples. Due to these constraints, it is not feasible to position any antenna directly facing the lower part of the jar, so we ultimately deploy six antennas arranged in an arch configuration, as illustrated in Fig. 1.

### The measurement procedure

The jars are actual commercial products. They are of elliptical cylindrical shape, whose minor axis is equal to $6.6\,\text {cm}$, a major axis of $8\,\text {cm}$, and a height of $10.5\,\text {cm}$. The contaminants exhibit a spherical shape with a diameter of $4\,\text {mm}$. We select plastic and glass as contaminants because they are expected to be present post-packaging in the food industry in case of contamination, and are hardly detectable with other systems^31^. We follow the same approach to conduct the measurements for both food materials, water, and oil. We arrange four plastic jars that shared the same shape and dimensions as the one illustrated in Fig. 1. Out of these four jars, we select two to be used as uncontaminated samples, while the remaining two are utilized to introduce various contaminants. The measurement process begins when the jar, carried by the conveyor belt, enters the area where our antenna array effectively emits radiation. The elements of the antenna array emit signals sequentially and receive them simultaneously as the jar is in motion. This operation enables the collection of signals at various positions of the jar and across different frequency points.

## Antenna design and testing

This section is a comprehensive description of the antenna design and operation. It includes the description of the design parameters, the impedance matching performance, and the experimental near-field evaluation. Furthermore, we evaluate the capability of detecting contaminants using the electric field (E-field) spatial coverage analysis and the time domain reflectometry.

### Antenna design parameters

**Figure 3:**
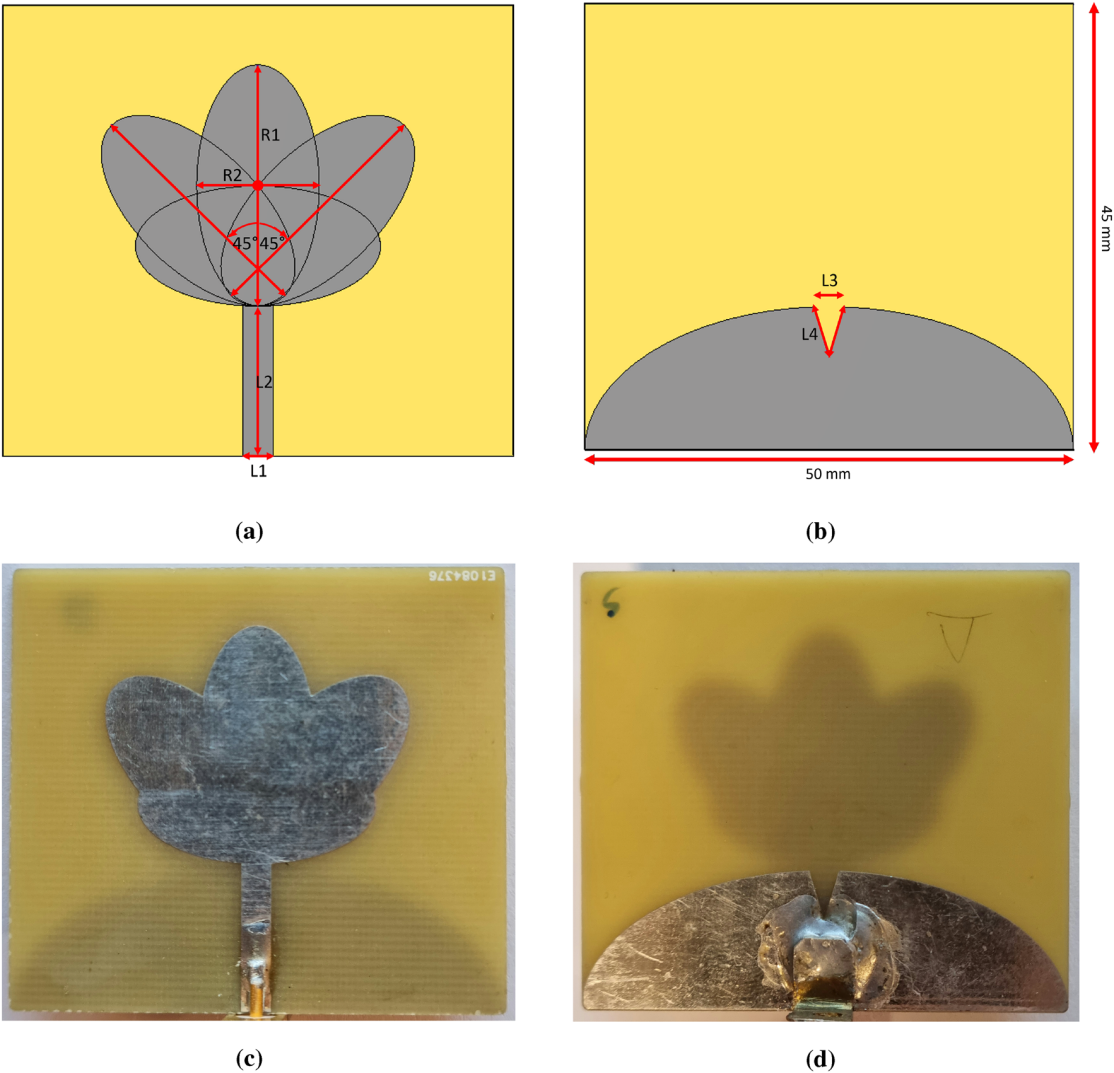
(**a**) Front view of the design, (**b**) rear view of the design, (**c**) front view after fabrication, (**d**) rear view after fabrication. Red labels indicate the following: L1 and L2 represent the width and length of the antenna’s transmission line, while R1 and R2 denote the longer and shorter radii of the four ellipses comprising the antenna structure. Additionally, L3 and L4 correspond to the dimensions of the V-cut triangular shape in the ground plane.

Selecting the appropriate antenna type is pivotal in designing an effective MW sensing system. Exploring the role of antennas in this context involves considering factors such as bandwidth, polarization, radiation pattern, and gain; however, given our focus on applying the sensing technique in the near-field region, our concern lies not on the gain. As stated in the system description section, we are using a PCB-printed monopole antenna with a flower shape, derived from^32^. Omni-directional antenna has a wide angular coverage, enabling it to emit and collect the retrieved scattering parameters from all directions. This capability facilitates the detection of contaminants at different positions within the jar. The simplicity and omnidirectional nature of the monopole antennas make them well-suited for close-range applications, however, obtaining a wide bandwidth antenna is a challenge in terms of radiation power stability across the entire bandwidth, impedance matching, and electrical size constraints. Taking into account all these parameters, we optimize the antenna to adapt to our specific application requirements, focusing on achieving the desired stability in the radiation pattern, the required bandwidth, and the impedance matching. The antenna is designed according to the following standards: the metallic flower structure of the antenna and the ground plane have a thickness of $0.03\,\text {mm}$ and are printed on a standard substrate of FR-4 ($\epsilon _r = 4.1$) with a thickness of $1.55\,\text {mm}$. The antenna primarily consists of four ellipses as shown in Fig. 3, each with a larger radius (R1) of $24\,\text {mm}$ and a smaller radius (R2) measuring $12\,\text {mm}$. The primary ellipse is aligned with the transmission line at a zero-degree angle relative to the line. Two additional ellipses contribute to forming the flower-shaped structure by being rotated +45^∘^ and $-45^\circ$ with respect to both the transmission line and the centrally positioned ellipse. The fourth ellipse is aligned horizontally and is rotated 90^∘^ with respect to the transmission line. The overall dimension of the antenna is $50\times 45 \,\text {mm}^2$. L1 and L2 represent the width and length of the transmission line, with values of $3\,\text {mm}$ and $15\,\text {mm}$, respectively. The ground plane has a semi-ellipsoidal shape with an optimal triangular cut, as illustrated in Fig. 3b. L3 and L4 are equal to $3\,\text {mm}$ and $5.2\,\text {mm}$ respectively and denote the optimal dimensions of the V-cut, which are determined for the best performance in terms of the impedance matching in the frequency band of interest.

The limit of the near-field region is determined by

2 $$\begin{aligned} \hspace{65mm} \text {r} < \frac{2\text {D}^2}{\lambda }, \end{aligned}$$

where $\text {D} = 6.7\,\text {cm}$ is the maximum dimension of the antenna (the diagonal), and $\lambda$ is the wavelength. In the selected frequency bands, we compute the near-field radiating radius (r) using Eq. (2) at $1.5\,\text {GHz}$ and $10\,\text {GHz}$, resulting in values of $4.5\,\text {cm}$ and $30\,\text {cm}$ respectively. In our setup, the antenna is about $5\,\text {cm}$ away from the jar. This implies that our measurements are conducted in the near-field region for oil-based products in the 6 to $10\,\text {GHz}$ bandwidth. While for water-based products, at lower frequencies within the 1.5 to $3.5\,\text {GHz}$ bandwidth, the jar is positioned at the edge of the near-field region. However, at the higher frequencies in this bandwidth, it falls within the near-field region. For a comprehensive understanding of the performance of the antenna array elements integrated with our sensing system, we conduct simulations and measurements to obtain the near-field. The upcoming subsections provide a thorough analysis and assessment of the propagation of the E-field radiated by the antenna in the region of interest.

### Impedance matching and near-field measurements

**Figure 4:**
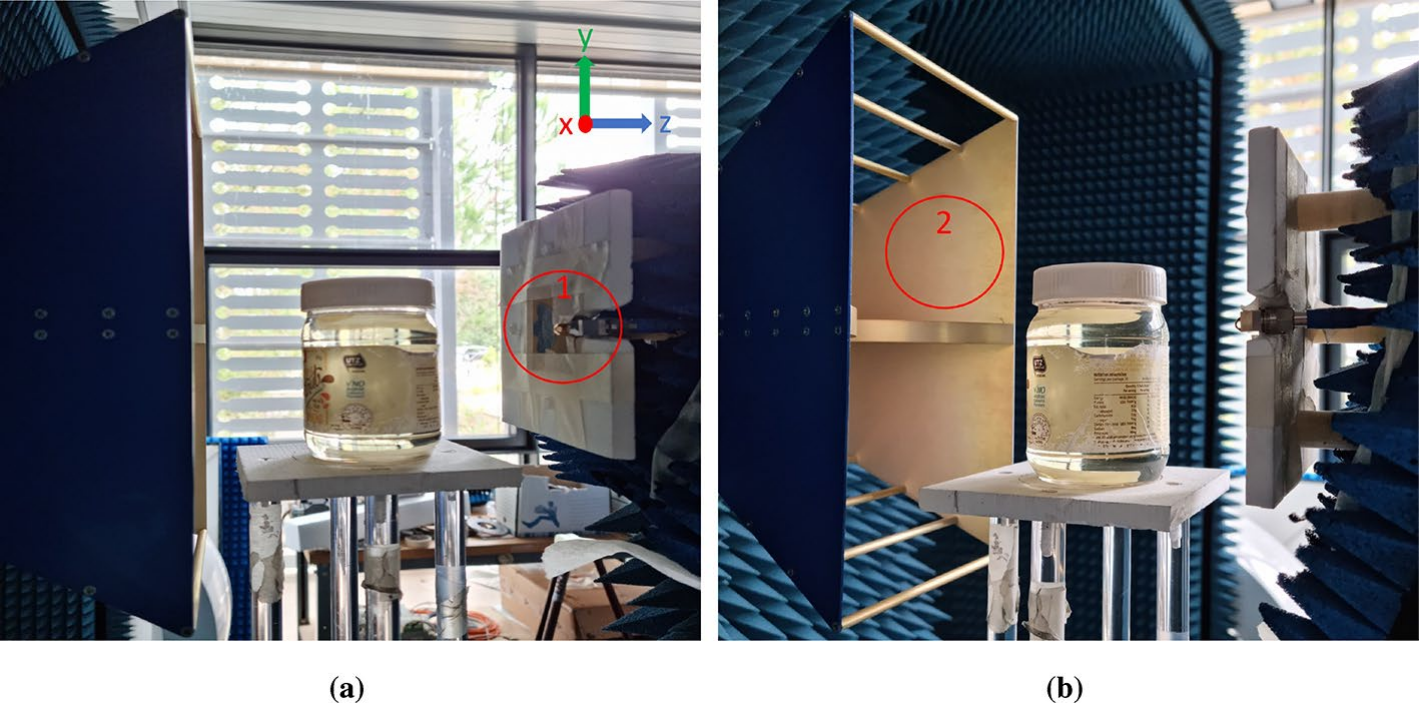
The system for near-field measurements. (**a**) The measurement setup displays the transmitting antenna, denoted as 1, which is the flower antenna used in the setup. (**b**) The measurement setup also features the receiving wideband horn antenna, labeled as 2.

The near-field measurement system, which is also used for impedance matching evaluation, is shown in Fig. 4. It comprises the network analyzer, the transmitting antenna employed in our study, and the receiving antenna, which is a wide-band horn antenna operating starting from $2\,\text {GHz}$. For the near-field measurements and later for the time-domain analysis, which are useful to fully understand and analyze the addressed problem, we conduct measurements and simulations across a broad bandwidth ranging from 2 to $12\,\text {GHz}$.

**Figure 5:**
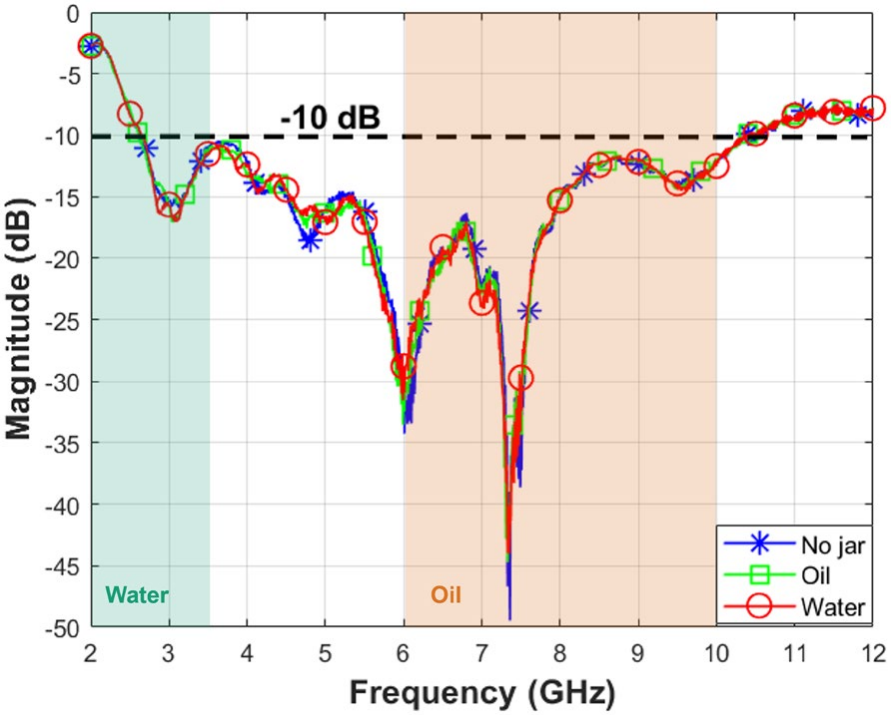
The measured amplitude of reflection coefficients for the realized antenna in three scenarios: with an empty jar, with a jar filled with oil, and with a jar filled with water.

For the impedance matching analysis, we measure the reflection coefficients at the antenna port. We investigate three scenarios that are reported in Fig. 5. The first one displays the impedance matching of the antenna in the absence of any jar (blue curve). This plot displays good matching performance in the bandwidths of interest except at the lowest frequencies of the lower bandwidth, as highlighted in green and orange shaded areas in Fig. 5. However, the real system operates in the presence of a jar, which is why we investigate two additional scenarios, one with a jar filled with oil (green curve), and the other with a jar filled with water (red curve). The three curves are almost superimposed, showing that the presence of the jar does not significantly affect the impedance matching. The antenna is overall well matched, except below 2.5 GHz. However, mismatching does not mean that no power is transmitted into the jar especially for water-based products, while the penetration depth is 6 cm at 2 GHz (from Fig. 2c). So it is too early to exclude the frequencies between 1.5 and 2.5 GHz at this stage, but we will investigate this frequency band during the analysis of the experiments and the classification results.

**Figure 6:**
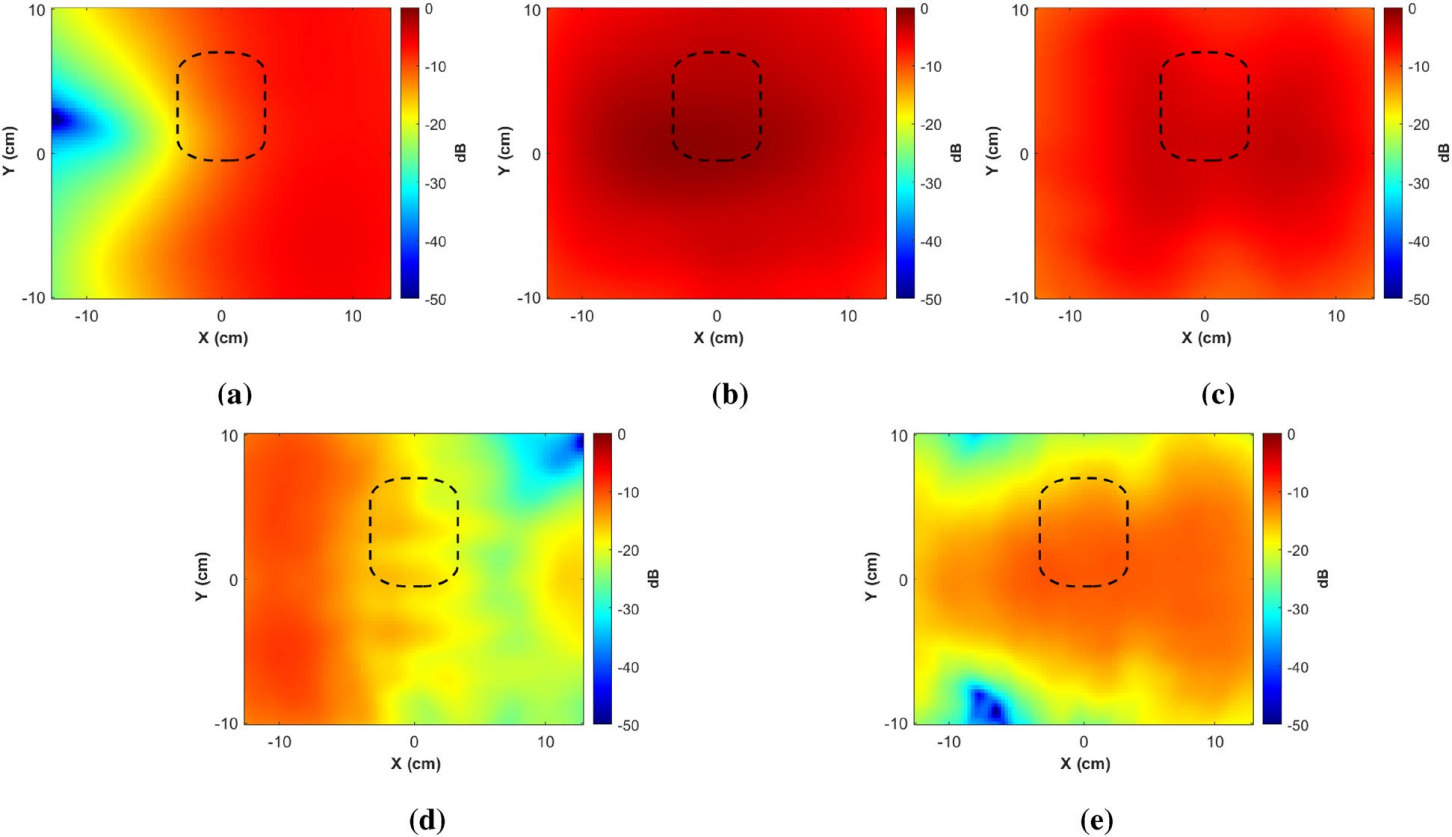
The measured amplitude of the antenna’s near-field (normalized to the maximum magnitude value across all frequency points) in the absence of any jar at: (**a**) 2 GHz, (**b**) 4 GHz, (**c**) 6 GHz, (**d**) 8 GHz, and (**e**) 10 GHz The rectangular shape featured in the plot indicates the anticipated location of the food jar within the measurement system.

Next, we measure the near-field of the antenna. For this, we make use of the mechanical stepper motor connected to the receiving horn antenna for aim scanning. The two antennas are centered with respect to each other and positioned $17\,\text {cm}$ apart. The horn antenna captures the radiated waves within a scanning area that extends $20\,\text {cm}$ in the vertical y-direction and $25\,\text {cm}$ in the horizontal x-direction with an incremental step of $2.5\,\text {mm}$ in both directions. Since we aim to measure the near-field of the antenna, measurements are conducted without the jar. The results are displayed in Fig. 6. The plots illustrate the magnitude of the near-field (normalized to the maximum magnitude value across all frequency points), illustrating a reduction in field strength with increasing frequency, especially beyond $8\,\text {GHz}$. This will be confirmed by the overall system measurements as described in the dataset construction section.

### E-field spatial coverage

**Figure 7:**
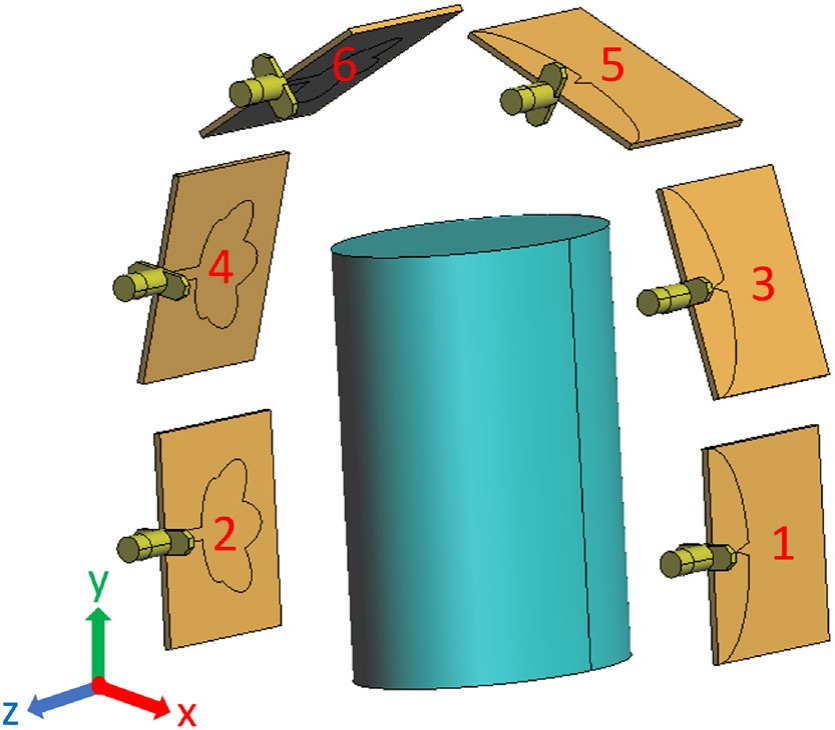
The design system used for E-field spatial coverage analysis.

The E-field spatial coverage can be analyzed as an indicator of how effectively an antenna array illuminates a specific point within the radiated volume. In simpler terms, it assesses how thoroughly the electromagnetic waves emitted by the antenna array reach and interact with a particular point within the tested space. For a better interpretation of E-field spatial coverage, we simulate the system in its real dimensions, as illustrated in Fig. 7. We choose the water case due to the challenge that high losses imply. The simulations are conducted at a frequency equal to $2.5 \,\text {GHz}$, which is the center frequency in the selected bandwidth for testing water jar samples. The E-field spatial coverage is defined as follows:

3 $$\begin{aligned} \hspace{55mm} C_E^\alpha (\textbf{r},f) = \frac{\sum _{n = 1}^T\mid \textbf{E}_n^\alpha (\textbf{r},f) \mid }{\max \sum _{n = 1}^T\mid \textbf{E}_n^\alpha (\textbf{r},f) \mid }, \end{aligned}$$

where $\textbf{r}$ is the positioning vector, *T* is the number of antennas, and $\textbf{E}_n^\alpha$ with $\alpha = i,t$ and *s*, is the incident, total, and scattered E-fields, respectively. Each $\textbf{E}_n^i$ is obtained with the *n*-th antenna radiating with the jar filled with water only, instead, the $\textbf{E}_n^t$ is the E-field radiated by the *n*-th antenna with contaminants inside the filled jar. Finally, $\textbf{E}_n^s = \textbf{E}_n^t - \textbf{E}_n^i$ represents the E-field scattered by the contaminants. When the *n*-th antenna is radiating, all the others are matched to 50 $\Omega$. Hence, $T = 6$ different simulations are performed to get the E-field spatial coverage with each time a different radiating antenna.

**Figure 8:**
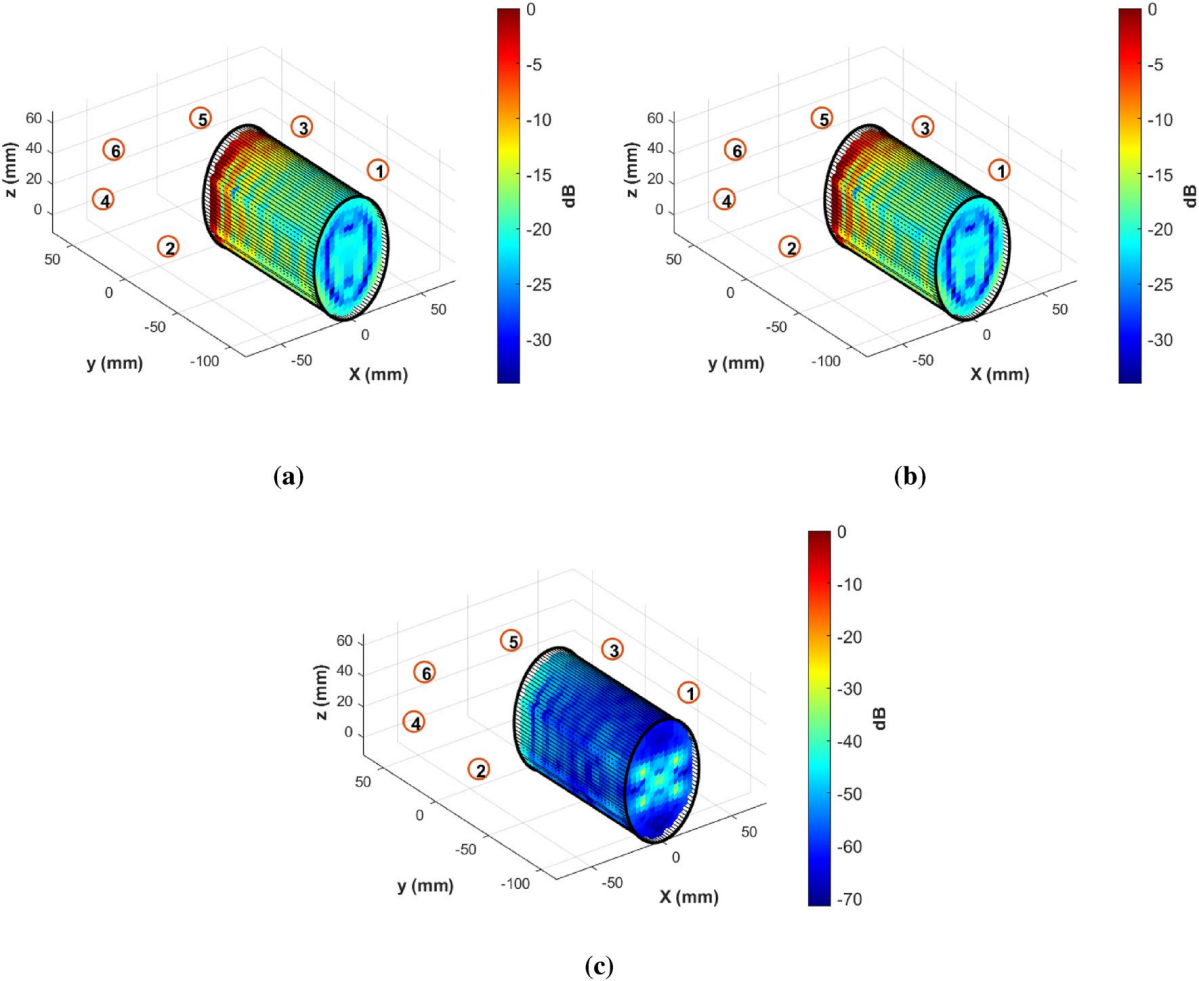
The E-field spatial coverage at 2.5 GHz inside the water jar: (**a**) incident, (**b**) total, and (**c**) scattered.

Figure 8a shows the $C_E^i(\textbf{r})$ and it is evident that we got the lowest E-field coverage at the bottom of the jar. Hence, to consider the worst scenario, the contaminants, which are five plastic spheres with 2 mm in diameter, are placed in the bottom of the jar. Then, Fig. 8b shows the $C_E^t(\textbf{r})$, which is the coverage when the contaminants are present: the total coverage appears very similar to the incident one. However, the scattered coverage shown in Fig. 8c clearly shows the presence of the contaminants at the accurate location, which are clearly distinguishable.

### Time-domain analysis

The time domain reflectometry is commonly used in non-destructive evaluation, showing that the reflection coefficients carry relevant information about the object under test; hence we use here to investigate our simulated and measured data. The objective of examining the time-domain response of the reflection coefficient parameters, obtained from both simulations and measurements, is to study the reflections of the signal emanating from the radiated region. This analysis can be conducted through direct time-domain measurements or by first performing a frequency-domain measurement followed by the Inverse Fast Fourier Transform (IFFT). To identify discontinuities within the system, a large time-domain observation window with a small interval is required. The reflection coefficients are measured across a frequency range of $2\,\text {GHz}$ to $12\,\text {GHz}$ at intervals of $10\,\text {MHz}$ for both the measurements and simulations. By comparing the electrical distance in both measurements and simulations across various media, along with evaluating the distance line ($\text {d}x$) obtained from the time domain analysis, we successfully detect the key reflection points within our system. These points include the antenna’s feeding port, its phase center, the jar filled with water, and most crucial the contaminant inside the jar.

**Figure 9:**
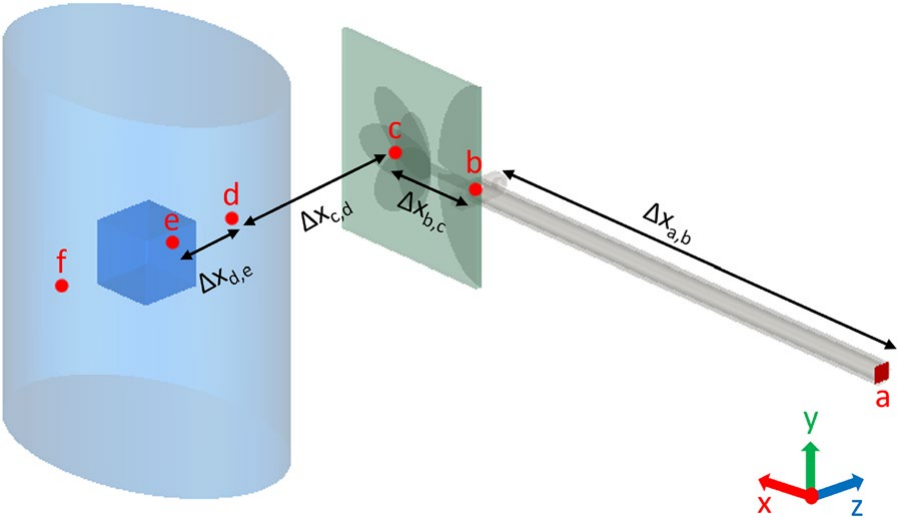
The simulation system similar to the measurements except for the presence of the foreign body.

In Fig. 9 we present the simulated system. The labels (a–f) within this figure correspond to the following points: (a) the waveguide port, (b) the feeding port of the antenna, (c) the center of the antenna structure, (d) the center of the face of the water jar opposite to the antenna, (e) the position of the contaminant inside the water, and (f) the edge of the jar from the second side. Four simulations are performed to analyze the time domain behavior under different scenarios. The first simulation is used as a reference, involving a jar filled with water without intrusions. In two of the remaining three simulations, a plastic cube contaminant is introduced, each with a different position within the jar. The last simulation involved changing the contaminant material to metallic (copper). The IFFT operation is applied to the simulated reflection coefficient data, and the distance line, $\text {d}x$, is calculated according to:

4 $$\begin{aligned} \hspace{60mm} \text {d}x = \frac{c\cdot \text {d}t}{2},\quad \text {d}t = \frac{1}{\text {d}f}, \end{aligned}$$

where $\text {d}x$ represents the distance line in centimeters, $c$ is the speed of light in free space, $\text {d}t$ is the time interval between consecutive points in the time domain, and $\text {d}f$ is the frequency resolution.

For more accurate calculations, it is important to define the range resolution as the antenna’s capability to distinguish between closely spaced targets. Discussing range resolution is important for addressing discontinuities in the antenna and the system. For example, to detect these reflection points accurately, the range resolution must be smaller than the distance between any two consecutive points in Fig. 9. Therefore, we need to consider the value of the range resolution in our calculations in determining the positions of these discontinuity points. The range resolution is defined as follows:

5 $$\begin{aligned} \hspace{65mm} \Delta {\text {R}} = \frac{c}{2\cdot \text {BW}}, \end{aligned}$$

where $\Delta {\text {R}}$ is the range resolution, and $\text {BW} = 10$ GHz is the bandwidth of the conducted simulations and measurements. According to Eq. (5), the range resolution is calculated to be 1.5 cm.

**Figure 10:**
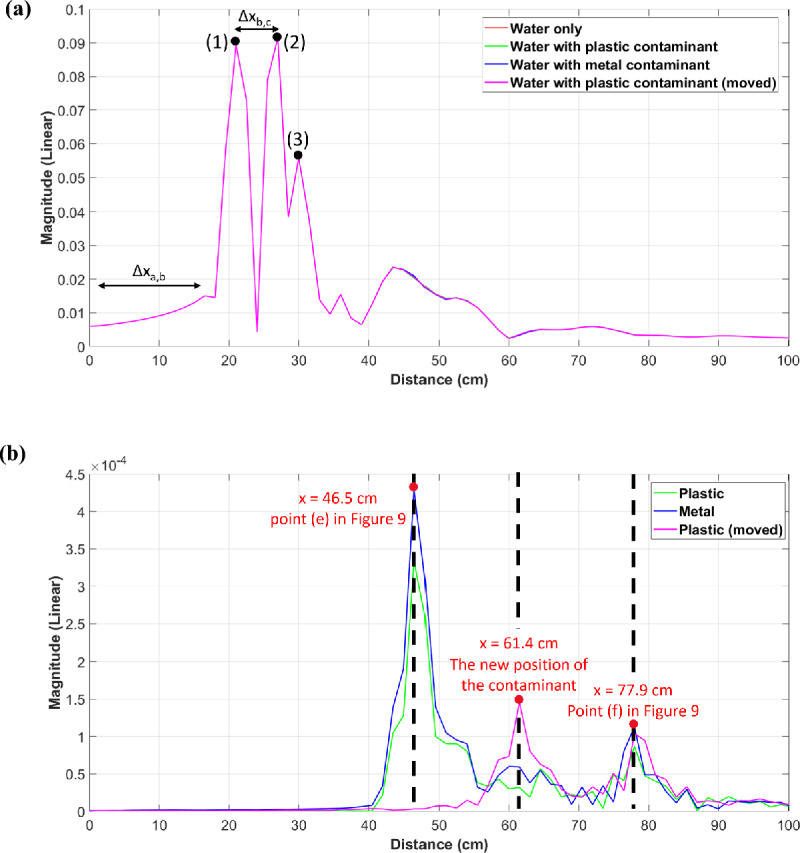
Time-domain analysis.

**Table 2:** Analysis of the distance $\Delta X$ as illustrated in Fig. 10.

	Relative permittivity ($\epsilon _r$)	Geometrical distance (cm)	Electrical distance theoretical (cm)	Electrical distance simulation (cm)
$\Delta X_{a,b}$	2.1	13.5	19.6	$18 \pm 1.5$
$\Delta X_{b,c}$	4.3	2.7	5.6	$6 \pm 1.5$
$\Delta X_{c,d}$	1	5.1	5.1	$3 \pm 1.5$
$\Delta X_{d,e}$	78	1.85	16.3	$16.4 \pm 1.5$
$\Delta X_{a,e}$	–	$\sum \Delta X (geometrical) = 23.15$	$\sum \Delta X (electrical) = 44.6$	$46.5 \pm 1.5$

The second column displays the relative permittivity of the medium along the specified distance; the third column shows the corresponding geometrical distance; the fourth column represents the corresponding electrical distance (theoretical); and the fifth column shows the electrical distance (simulation) that appears in Fig. 10a, b.

**Figure 11:**
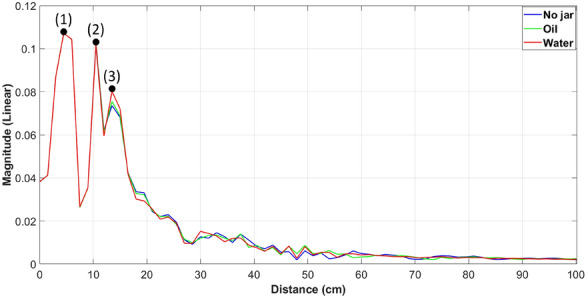
The amplitude of the IFFT results for the measured reflection coefficients.

Figure 10a shows the IFFT results for the simulated reflection coefficients obtained at point a in Fig. 9. The labels (1), (2), and (3) within this subfigure correspond to the reflection points b, c, and e indicated in Fig. 9, respectively. The positions of these peaks on the IFFT plots are determined by calculating the electrical distance by multiplying the geometrical distance (obtained from the simulated design or the measurement system) by the square root of the relative permittivity of the propagating medium, as shown in Table 2. This allows us to discern which part of the conducted simulation or real measurements corresponds to each peak in the IFFT plot. In Fig. 10a, we are unable to detect the presence of the contaminant inside the jar, as the plots are superimposed with the reference curve (the one without the contaminant). To address this issue, we subtract the recorded reflection coefficients simulated when the intrusion is present from that of the reference simulation. Then, we perform the IFFT on the remaining values to see if we can identify the positions of the contaminants along the distance line. Figure 10b displays the outcomes of this approach, and it is evident that the peak obtained at $x = 46.5\,\text {cm}$ is a result of the contaminant presence within the water. This peak is notably more pronounced when the contaminant is metallic. The displacement of the contaminant within the jar influences the location of the reflection point indicated by a peak at $x = 61.4\,\text {cm}$, while the peak at $x = 77.9\,\text {cm}$ arises from the reflection of the rear side of the jar. This peak appears as a result of the presence of the contaminant within the water, influencing the propagation path of the wave within the medium. This deviation from the reference scenario, where no contaminant is present inside the jar, causes the observed difference at the third peak in Fig. 10b.

Comparing the data in the final two columns of Table 2 reveals a good coherency between the theoretically estimated reflection points positions and those evident in the IFFT plot illustrated in Fig. 10a, b, taking into account the range resolution. The error observed in the ($\Delta X_{c,d}$) can be attributed to the assumed exact position of the antenna’s phase center at point c, as depicted in Fig. 9.

To further investigate the system’s ability to detect changes in the reflection coefficients based on the jar contents through measurements, we record the antenna’s reflection coefficient parameters using the same system previously employed in the near-field study (shown in Fig. 4). We explore three distinct scenarios: one without any jar, another with a jar filled with oil, and a third with the same jar filled with water, but this time without the need for scanning. In Fig. 11, the labels displayed on the curve represent the estimated reflection points. The peaks marked with (1) and (2) correspond to the feeding port and the phase center of the antenna, respectively. The three curves overlap until reaching the peak at point (3), which indicates the reflection point originating from both the support holding the jar and the jar itself, as shown in Fig. 4. The reflections at this point distinctly demonstrate the influence of the entire medium within the jar on the reflection intensity. Specifically, it is more pronounced when the jar is filled with water (the red curve) compared to when it contains oil (the green curve) or the scenario where reflections are only due to the support without the presence of a jar (the blue curve). It is noteworthy to mention the consistency of the three highlighted peaks (1), (2), and (3) in both plots, the simulation in Fig. 10a, and the measurements in Fig. 11. However, the position of these points is shifted by the length of the coaxial cable ($\Delta X_{a,b}$) in Fig. 10a.

## Measurements and classification

### Measurements and settings

**Figure 12:**
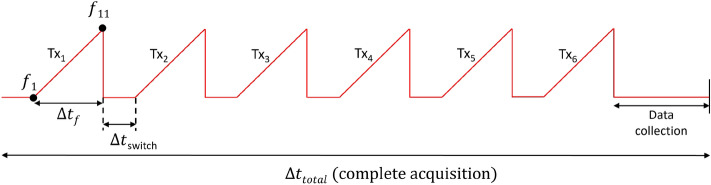
The time frame of one complete data acquisition (one single measurement).

After determining the two frequency bands of interest, we have to set up the number of frequency points within these bands. This directly depends on the speed of the conveyor belt, which is equal to 33 cm/s, and on the maximum allowed acquisition time, required for the measurement of one sample jar, which corresponds to around 50 ms, and is indicated with $\Delta t_{total}$ in Fig. 12. This acquisition time includes the starting time of the measurements, and six periods of $\Delta t_{f}$, where each period represents a frequency ramp, which is the time needed for a gradual frequency increase between the minimum and maximum frequency points within the selected frequency bands. Additionally, there are 5 $\Delta t_{switch}$ periods, which corresponds to the switching time between the 6 transmitting antennas, and finally, the time needed to collect the data. Choosing the number of frequency points is a trade-off between having frequency diversity from the measurements and the time based on the moving jar carried by the conveyor belt. A higher number of frequency points provides more detailed information but may require longer measurement times, which makes the process inapplicable. $\Delta t_{f}$ is defined as:

6 $$\begin{aligned} \hspace{65mm} \Delta t_{f} = N_f \cdot t_{IF}, \end{aligned}$$

where $N_f$ is the number of the frequency points and $t_{IF}$ is the time taken by the VNA to sweep through the specified frequency bandwidth and collect data points, and it is inversely proportional to the intermediate frequency filter of the VNA (*IF*). We aim for a small value of *IF* for achieving measurements with low noise. However, a small *IF* value leads to a large $t_{IF}$ value, indicating a preference for a reduced number of frequency points ($N_f$). Based on this analysis, we have found that recording data at $N_f = 11$ frequency points in the two bands with $IF = 1$ kHz is compatible with our requirements.

To obtain 11 frequency points across the two frequency bands, we must set the frequency step within each band. For water-based food jars, a step of $200\,\text {MHz}$ is used in the $1.5\,\text {GHz}$ to $3.5\,\text {GHz}$ band, while for oil-based jars, a $400\,\text {MHz}$ step is set in the $6\,\text {GHz}$ and $10\,\text {GHz}$ frequency band.

In Fig. 12, it is evident that the transition between antennas ($Tx_i$), where *i* represents the index of the radiated antenna, occurs once the preceding antenna has completed its operation over the 11 frequencies in the bands. At each time when an antenna radiates, the other 5 antennas and the radiated antenna receive the retrieved scattered waves. After each acquisition, we obtain a set of 11 matrices at the 11 frequency points, each comprising 36 elements. These matrices contain valuable information regarding the condition of the food product being tested. Moreover, we underline that, during the measurements, the jar is in motion as the transition between antennas and frequency sweeping takes place. Therefore, the obtained matrices are not symmetrical, since the dynamic movement of the jar leads to changes in the scattering behavior of the MW.

### Dataset construction

To generate the datasets, we perform multiple repetitions of measurements (illustrated in Table 3), spanning hundreds of times for each specific case. The incorporation of diverse data collection approaches, such as conducting measurements on different days, swapping between the contaminated and uncontaminated jars, and slightly rotating the jar before each single measurement, plays a crucial role in enhancing the robustness of our findings.

**Table 3:** The number of measurements conducted with oil and water jar samples containing various types of contaminants.

Scenarios	Water jar	Oil jar
No contaminants	300	230
PTFE	100	130
SLG	100	130

An externally triggered script is used to control the desired parameters in our measurements. This script enables us to conduct uninterrupted hundreds of measurements while sequentially recording the scattering matrices. Every single measurement includes 11 matrices, each of size $6\times 6$. These 11 matrices are arranged in columns, as illustrated below:

7 $$\begin{aligned} \hspace{40mm} [S_{n}^{\mathbb {C}}] = \begin{bmatrix} S_{11,n}^{f_1} &{} S_{11,n}^{f_2} &{} \cdots &{} \cdots &{} S_{11,n}^{f_{10}} &{} S_{11,n}^{f_{11}} \\ S_{12,n}^{f_1} &{} S_{12,n}^{f_2} &{} \cdots &{} \cdots &{} S_{12,n}^{f_{10}} &{} S_{12,n}^{f_{11}} \\ \vdots &{} \vdots &{} \vdots &{} \vdots &{} \vdots &{} \vdots \\ S_{16,n}^{f_1} &{} S_{16,n}^{f_2} &{} \cdots &{} \cdots &{} S_{16,n}^{f_{10}} &{} S_{16,n}^{f_{11}} \\ S_{21,n}^{f_1} &{} S_{21,n}^{f_2} &{} \cdots &{} \cdots &{} S_{21,n}^{f_{10}} &{} S_{21,n}^{f_{11}} \\ \vdots &{} \vdots &{} \vdots &{} \vdots &{} \vdots &{} \vdots \\ S_{26,n}^{f_1} &{} S_{26,n}^{f_2} &{} \cdots &{} \cdots &{} S_{26,n}^{f_{10}} &{} S_{26,n}^{f_{11}} \\ \vdots &{} \vdots &{} \vdots &{} \vdots &{} \vdots &{} \vdots \\ \vdots &{} \vdots &{} \vdots &{} \vdots &{} \vdots &{} \vdots \\ S_{66,n}^{f_1} &{} S_{66,n}^{f_2} &{} \cdots &{} \cdots &{} S_{11,n}^{f_{10}} &{} S_{66,n}^{f_{11}} \\ \end{bmatrix} \end{aligned}$$

where each element $S_{ij,n}^{f_m}$ is a complex number and *n* refers to the index of the measurement sample, *i* and *j* are the indices corresponding to receiving and radiating antennas, respectively, and $f_m$ with $m = 1,\ldots ,11$ denotes the 11 frequency points. From Eq. (7), the following vectors are derived for each frequency $f_m$:

8 $$\begin{aligned}{} & {} \hspace{25mm} [S_{n}^{A,f_m}] = { \begin{bmatrix} |S_{11,n}^{f_m}|&\cdots&|S_{16,n}^{f_m}|&|S_{21,n}^{f_m}|&\cdots&|S_{26,n}^{f_m}|&\cdots&\cdots&|S_{61,n}^{f_m}|&|S_{66,n}^{f_m}| \end{bmatrix}^T} \end{aligned}$$

9 $$\begin{aligned}{} & {} \hspace{10mm} [S_{n}^{f_m}] = \begin{bmatrix} \begin{aligned} \mathcal {R}(S_{11,n}^{f_m})&\mathcal {I}(S_{11,n}^{f_m})&\hspace{-5pt}\cdots&\hspace{5pt}\mathcal {R}(S_{16,n}^{f_m})&\hspace{-10pt}\mathcal {I}(S_{16,n}^{f_m})&\hspace{5pt}\cdots&\mathcal {R}(S_{61,n}^{f_m})&\mathcal {I}(S_{61,n}^{f_m})&\hspace{-5pt}\cdots&\hspace{5pt}\mathcal {R}(S_{66,n}^{f_m})&\hspace{-10pt}\mathcal {I}(S_{66,n}^{f_m}) \end{aligned} \end{bmatrix}^T \end{aligned}$$

where A in Eq. (8) refers to the amplitude of the measured scattering parameters, while Eq. (9) contains both their real and imaginary parts in a separate form. Gathering the 11 vectors corresponding to the 11 frequencies allows us to obtain the following vectors:

10 $$\begin{aligned}{} & {} \hspace{40mm} [S_{n}^A] = { \begin{bmatrix} [S_{n}^{A,f_{1}}]&\hspace{6pt}[S_{n}^{A,f_{2}}]&\cdots&\cdots&\cdots&\hspace{6pt}[S_{n}^{A,f_{11}}] \end{bmatrix}^T} \end{aligned}$$

11 $$\begin{aligned}{} & {} \hspace{43mm} [S_{n}] = { \begin{bmatrix} [S_{n}^{f_{1}}]&\hspace{6pt}[S_{n}^{f_{2}}]&\cdots&\cdots&\cdots&\hspace{6pt}[S_{n}^{f_{11}}] \end{bmatrix}^T} \end{aligned}$$

which encompasses all the extracted scattering parameters from a tested sample. Each vector comprises either 396 entries for an amplitude-only dataset or 792 entries for a dataset with complex numbers data. With the provided vectors, we can generate the required datasets for both training and testing phases by collecting the complete set of vectors associated with all measurement samples in the following manner:

12 $$\begin{aligned}{} & {} \hspace{45mm} [S^A] = { \begin{bmatrix} [S_{1}^{A}]&\hspace{6pt}[S_{2}^{A}]&\cdots&\cdots&\cdots&\hspace{6pt}[S_{N}^{A}] \end{bmatrix}} \end{aligned}$$

13 $$\begin{aligned}{} & {} \hspace{46mm} [S] = { \begin{bmatrix} [S_{1}]&\hspace{6pt}[S_{2}]&\cdots&\cdots&\cdots&\hspace{6pt}[S_{N}] \end{bmatrix}} \end{aligned}$$

where N represents the total number of conducted measurements.

**Figure 13:**
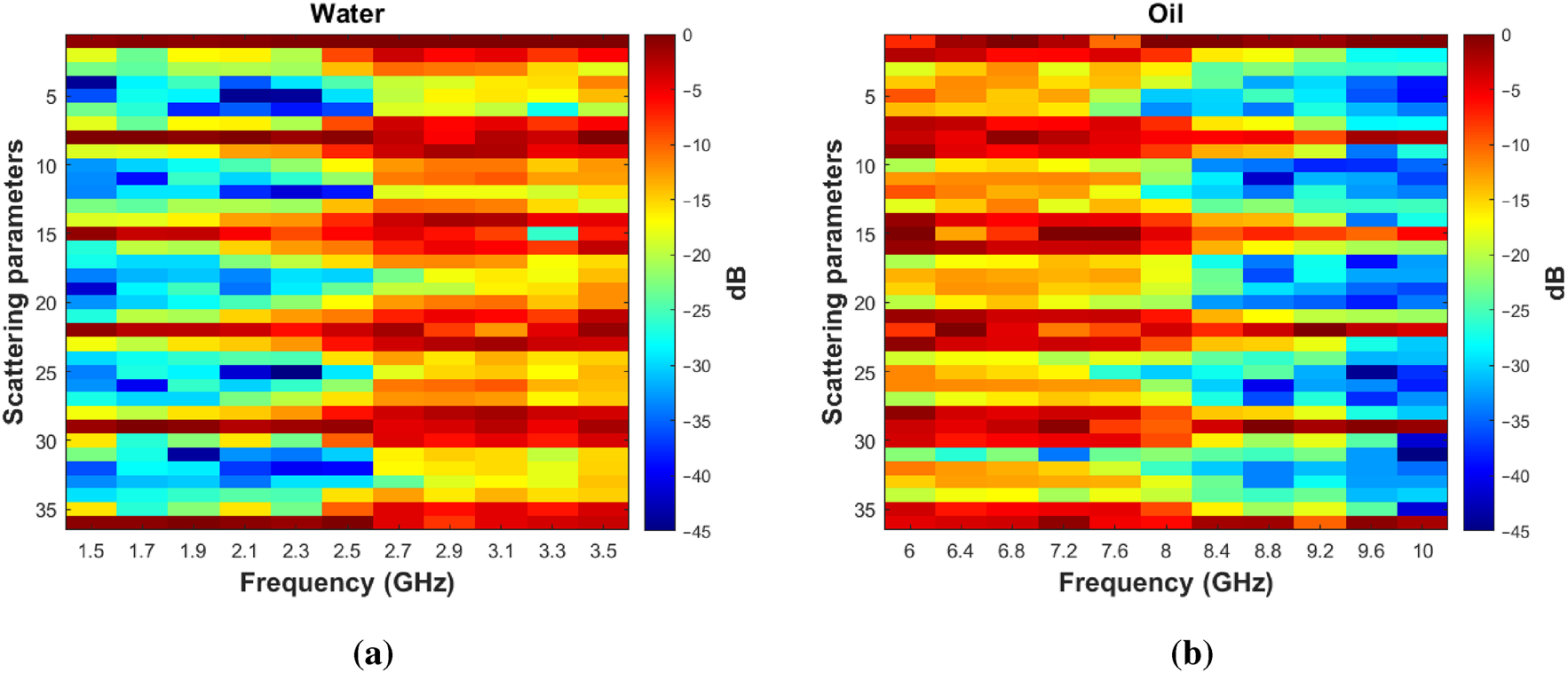
The normalized amplitude of the scattering matrix $[S_{n}]$ for two uncontaminated samples under test: (**a**) water, (**b**) oil.

Figure 13 displays the amplitude plot for two matrices $[S_{n}]$ corresponding to different test samples and normalized to the maximum amplitude value in each sample case. The magnitude values in Fig. 13a demonstrate better transmission performance of the employed antennas above $2.5\,\text {GHz}$ in the case of the water jar. This is coherent with the behavior of the antenna reflection coefficients in Fig. 5, where below 2.5 GHz the antenna is not well matched. Instead in Fig. 13b, the values indicate better transmission performance from $6 \,\text {GHz}$ up to $8\,\text {GHz}$, while, beyond that range, there is a decrease in the magnitude of the transmission coefficients. This behavior is related to the antenna near-field shown in Fig. 6 where there is an evident reduction of the radiated field above 8 GHz. The significant variation in the magnitude of transmission coefficients within the chosen frequency bands for oil-based jar samples and water-based jar samples enables us to selectively focus on subranges with favorable transmission behavior. The selective process within the frequency bands will be demonstrated in the experimental results section when we attempt classification based solely on the most significant magnitude for the transmission coefficients across these frequency bands.

### Support vector machine (SVM)

Employing machine learning tools in these applications becomes essential due to the critical requirement of categorizing samples from food jars as contaminated or uncontaminated within a production chain that operates in real-time. The power and the effectiveness of using such tools for classification arise from their remarkable efficiency in rapidly making decisions without the need for extensive data preprocessing. In this section, we explore the reasons for selecting the SVM algorithm for the classification procedure.

The SVM serves as a supervised binary classifier, renowned for its robustness and precision in ML algorithms. This is particularly evident when dealing with small datasets because SVM focuses on finding the best possible separation between classes by identifying support vectors, which are the data points closest to the decision boundary (the hyperplane). This characteristic allows SVM to make effective decisions in scenarios where the dataset size is limited as in our case. SVM comes in two primary categories: linear SVM and non-linear SVM. The first is preferable in straightforward classification problems when the data from both classes can be linearly separated. On the other hand, the second, non-linear SVM, is more suitable for scenarios where the data are non-linearly separable and correlated. In both cases, SVM operates by identifying the optimal hyperplane that effectively categorizes the desired classes for classification. The effectiveness of SVM hinges on the accurate tuning of its hyperparameters, one of which is denoted as “C”. This parameter plays a crucial role in adjusting the margin that separates the two classes. Regardless of whether linear or non-linear SVM is employed, selecting the appropriate value for “C” is essential. In non-linear SVM, a technique known as the “Kernel” function is used to discover the optimal hyperplane. This involves projecting the data into a higher-dimensional space, aiming to determine a hyperplane that effectively separates these data points. However, using the Kernel trick necessitates the calculation of additional hyperparameters. In our study, we use the Radial Basis Function (RBF) Kernel^33^, which introduces another important hyperparameter called Gamma ($\gamma$). Determining $\gamma$ holds significance, as it determines the weight assigned to samples based on their distance from the origin of the training dataset. Selecting appropriate values for both C and $\gamma$ is not a straightforward task. To address this challenge, we employ the Grey Wolf Optimizer^34^ (GWO) to systematically search for the optimal values of these two parameters. To explore further the implementation details of the classifier and the GWO method, we follow the same procedures outlined in^35^.

**Figure 14:**
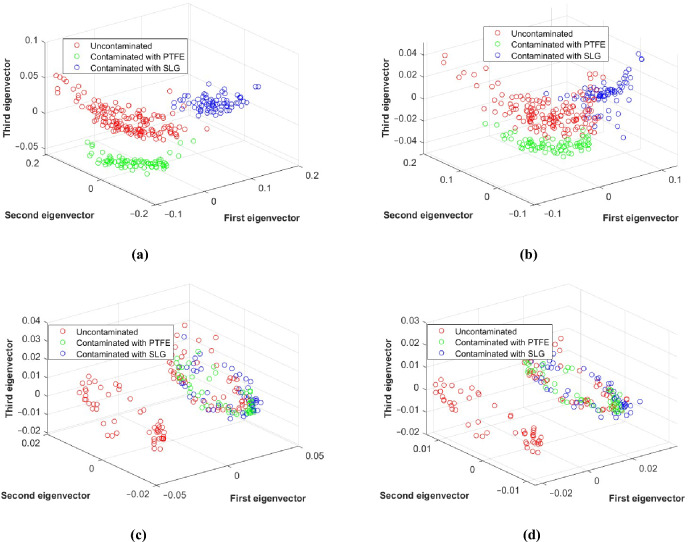
Projection of PCA results onto the three most significant eigenvectors. (**a**) Complex nature dataset (Water). (**b**) Amplitude-only dataset (Water). (**c**) Complex nature dataset (Oil). (**d**) Amplitude-only dataset (Oil).

The Principal Component Analysis (PCA) provides us with essential information regarding our dataset distribution, allowing visualization in lower dimensions through the variance of the most significant eigenvectors^36,37^. We applied PCA on different types of training sets to both water-based and oil-based food samples, including datasets with only amplitude values and datasets that encompass the complex nature of the S-parameters. Figure 14 illustrates the projection of the three most significant eigenvectors from the PCA outcomes. The data exhibit overlaps and demonstrates a markedly nonlinear relation between the contaminated and the uncontaminated samples in both magnitude-only and complex nature datasets for oil-based samples. On the other hand, for water-based samples, the magnitude-only dataset also displays overlaps, whereas the complex nature dataset shows linear separability and lack of correlation. Given this analysis of the plots, we investigated the possibility of employing nonlinear algorithms (SVM) that can effectively separate and categorize all our samples.

## Experimental results

Within this section, we provide the results derived after applying SVM non-linear classification algorithm to the datasets previously discussed in the preceding section. The outcomes acquired pertain to the testing of two categories of food products: those that are oil-based and those that are water-based. The two upcoming subsections present comprehensive findings, encompassing various factors that can influence the performance of the ML classifier. These factors include the complex numbers dataset, inserting only the amplitude part of the measurement data in the dataset, division of dataset portions (training and test sets), different frequency bands selection, and distinct types of classifications. The performance accuracies that appear in the following tables represent the average of over 100 classification trials. In each iteration of the algorithm, our dataset undergoes shuffling, and distinct samples are selected for both training and testing, while maintaining the same hyperparameters. Additionally, the confusion matrices included correspond to the worst reproducible obtained from the 100 algorithm runs.

Before presenting our results, it is important to establish the definition of the confusion matrix, a matrix summarizing classification outcomes. Table 4 displays the confusion matrix that includes the following entries: the number of true positive (TP), indicating uncontaminated cases correctly classified as uncontaminated; the number of false positive (FP), denoting uncontaminated cases misclassified as contaminated; the number of false negative (FN), representing contaminated cases misclassified as uncontaminated; and the number of true negative (TN), indicating contaminated samples correctly classified as contaminated cases. It is important to note that the number of FN is the critical parameter, as it indicates a flaw in the detection of contaminants.

**Table 4:** The confusion matrix.

	Positive	Negative
Positive	TP	FP
Negative	FN	TN

Moreover, the accuracy reported in the following tables is defined as follows:

14 $$\begin{aligned} \hspace{55mm} \text {Accuracy} = \frac{\text {TP} + \text {TN}}{\text {TP}+\text {TN}+\text {FP}+\text {FN}}\cdot 100 \end{aligned}$$

In the following subsections, we will analyze binary classifications, simply identifying contaminated and uncontaminated products, and multi-class classifications, where instead we are able to identify the contaminants type. In the multi-class analysis, three classes are considered: uncontaminated, contaminated with PTFE, and contaminated with SLG. Although the industry is interested in detecting whether their products are contaminated or not (binary classification), the multi-class classification provides the prevalence of the contaminants. This is important for identifying the part of the production chain that is responsible for the defects.

### Water-based contents

In this subsection, we present and discuss the accuracy percentages obtained from the classification results for the complex number dataset of water-based food products under different conditions shown in Table 5. The conditions include variations in the dataset split, type of classification, and the frequency band. Considering these conditions, we analyze and compare the obtained results. Table 5 presents results for two different dataset splits, that are 300–200 and 200–300 referring to the training and test portions samples of our dataset. The classification accuracy comes perfect in all binary classifications regardless of all the different conditions. In multiclass classifications, the accuracy remains consistently high across the different dataset splits, ranging from 99.8% to 100%. These results show that the performance of the used Ml model is robust and not significantly affected by the specific distribution of training and test samples. The minor errors that appear in the last two confusion matrices in Table 5 correspond to a single test sample that was contaminated with a small PTFE sphere. In the first error, it was classified as a contaminated case but with an SLG sphere, while in the second error, it was classified as an uncontaminated case, which is considered a more critical fault.

**Table 5:** Classification results on complex numbers dataset nature for water.

Dataset split (Training-test)	Type of classification	Frequency band [start : step : end] GHz	Confusion Matrices (On Test Set)	Accuracy (%)
300–200 samples	Binary	[1.5 : 0.2 : 3.5]	$$\left[ \begin{array}{cc} 150 &{} 0 \\ 0 &{} 50 \end{array}\right]$$	100
300–200 samples	Binary	[2.5 : 0.2 : 3.5]	$$\left[ \begin{array}{cc} 150 &{} 0 \\ 0 &{} 50 \end{array}\right]$$	100
200–300 samples	Binary	[1.5 : 0.2 : 3.5]	$$\left[ \begin{array}{cc} 200 &{} 0 \\ 0 &{} 100 \end{array}\right]$$	100
200–300 samples	Binary	[2.5 : 0.2 : 3.5]	$$\left[ \begin{array}{cc} 200 &{} 0 \\ 0 &{} 100 \end{array}\right]$$	100
300–200 samples	Multiclass	[1.5 : 0.2 : 3.5]	$$\left[ \begin{array}{ccc} 150 &{} 0 &{} 0 \\ 0 &{} 25 &{} 0 \\ 0 &{} 0 &{} 25 \end{array}\right]$$	100
300–200 samples	Multiclass	[2.5 : 0.2 : 3.5]	$$\left[ \begin{array}{ccc} 150 &{} 0 &{} 0 \\ 0 &{} 25 &{} 0 \\ 0 &{} 0 &{} 25 \end{array}\right]$$	100
200–300 samples	Multiclass	[1.5 : 0.2 : 3.5]	$$\left[ \begin{array}{ccc} 200 &{} 0 &{} 0 \\ 0 &{} 49 &{} 1 \\ 0 &{} 0 &{} 50 \end{array}\right]$$	99.8
200–300 samples	Multiclass	[2.5 : 0.2 : 3.5]	$$\left[ \begin{array}{ccc} 200 &{} 0 &{} 0 \\ 1 &{} 49 &{} 0 \\ 0 &{} 0 &{} 50 \end{array}\right]$$	99.8

Using only the amplitude part of the scattering parameters for the classification process has practical advantages, especially for implementation in an in-line production chain. It simplifies the system by requiring a power measurement system that captures magnitude without the need for measuring the phase. In Table 6, we present the results acquired from a dataset derived exclusively from the magnitude component of the measurement samples. For binary classification approach, in the classification performed on 1.5 to 3.5 GHz frequency band, both dataset splits achieved 100% accuracy, with no misclassifications. While, from 2.5 to 3.5 GHz frequency band, high accuracy results recorded 99.7% for the two dataset splits with only a few misclassifications recorded in the confusion matrices for one uncontaminated sample classified as contaminated. On the other hand, for the multiclass classification, in the frequency band between 1.5 and 3.5 GHz, the 300-200 dataset split achieved a high accuracy of 99.9% with minor misclassifications indicated in the confusion matrices as an uncontaminated test sample recognized as contaminated. While, in 2.5-3.5 GHz frequency band, the accuracy was slightly lower at 99.5%, with a small number of misclassifications obtained. The second dataset split for both frequency bands achieved a high accuracy of 99.8%, with similar confusion matrices, which is a sample contaminated with PTFE classified as a sample contaminated with SLG. In summary, Table 6 demonstrates that the classification experiments using magnitude-only data for water achieved high accuracies, indicating the effectiveness of the approach. The results are consistent across different dataset splits and frequency bands, with minor variations in accuracy and misclassifications.

**Table 6:** Classification results on only amplitude dataset nature for water.

Dataset split (Training-test)	Type of classification	Frequency band [start : step : end] GHz	Confusion matrices (on test set)	Accuracy (%)
300–200 samples	Binary	[1.5 : 0.2 : 3.5]	$$\left[ \begin{array}{cc} 150 &{} 0 \\ 0 &{} 50 \end{array}\right]$$	100
300–200 samples	Binary	[2.5 : 0.2 : 3.5]	$$\left[ \begin{array}{cc} 149 &{} 1 \\ 0 &{} 50 \end{array}\right]$$	99.7
200–300 samples	Binary	[1.5 : 0.2 : 3.5]	$$\left[ \begin{array}{cc} 200 &{} 0 \\ 0 &{} 100 \end{array}\right]$$	100
200–300 samples	Binary	[2.5 : 0.2 : 3.5]	$$\left[ \begin{array}{cc} 199 &{} 1 \\ 0 &{} 100 \end{array}\right]$$	99.7
300–200 samples	Multiclass	[1.5 : 0.2 : 3.5]	$$\left[ \begin{array}{ccc} 149 &{} 1 &{} 0 \\ 0 &{} 25 &{} 0 \\ 0 &{} 0 &{} 25 \end{array}\right]$$	99.9
300–200 samples	Multiclass	[2.5 : 0.2 : 3.5]	$$\left[ \begin{array}{ccc} 150 &{} 0 &{} 0 \\ 0 &{} 24 &{} 1 \\ 0 &{} 0 &{} 25 \end{array}\right]$$	99.5
200–300 samples	Multiclass	[1.5 : 0.2 : 3.5]	$$\left[ \begin{array}{ccc} 200 &{} 0 &{} 0 \\ 0 &{} 49 &{} 1 \\ 0 &{} 0 &{} 50 \end{array}\right]$$	99.8
200–300 samples	Multiclass	[2.5 : 0.2 : 3.5]	$$\left[ \begin{array}{ccc} 200 &{} 0 &{} 0 \\ 0 &{} 49 &{} 1 \\ 0 &{} 0 &{} 50 \end{array}\right]$$	99.8

### Oil-based contents

Similarly to how we presented the classification results for water-based samples, we are now presenting the results for classifying food samples based on oil content. For the binary classification on complex nature dataset, in the 6 to 10 GHz frequency band with a 250–240 dataset split, an accuracy of 96.7% is achieved. The confusion matrix shows a high number of correctly classified samples with eight false positive recorded. While for the classification over 6 to 8 GHz frequency band with the same dataset split, the accuracy slightly increases to 98% and the confusion matrix indicates a lower number of false positives (4 samples). For the multiclass classification experiments, 95% and 95.5% performance accuracies are reported for the 290-200 dataset split respectively on the two frequency bands. The confusion matrices reveal significant misclassifications across the uncontaminated class. In summary, Table 7 manifests varying results across different classification scenarios. The binary classification experiments achieved high accuracy in both frequency bands, while the multiclass classification experiments exhibited lower but still effective accuracy.

**Table 7:** Classification results on complex numbers dataset nature for oil.

Split (training-test)	Type of classification	Frequency band [start : step : end] GHz	Confusion matrices (on test set)	Accuracy (%)
250–240 samples	Binary	[6 : 0.4 : 10]	$$\left[ \begin{array}{cc} 72 &{} 8 \\ 0 &{} 160 \end{array}\right]$$	96.7
250–240 samples	Binary	[6 : 0.4 : 8]	$$\left[ \begin{array}{cc} 76 &{} 4 \\ 0 &{} 160 \end{array}\right]$$	98
290–200 samples	Multiclass	[6 : 0.4 : 10]	$$\left[ \begin{array}{ccc} 90 &{} 10 &{} 0 \\ 0 &{} 50 &{} 0 \\ 0 &{} 0 &{} 50 \end{array}\right]$$	95
290–200 samples	Multiclass	[6 : 0.4 : 8]	$$\left[ \begin{array}{ccc} 91 &{} 9 &{} 0 \\ 0 &{} 50 &{} 0 \\ 0 &{} 0 &{} 50 \end{array}\right]$$	95.5

In Table 8, in the binary classification scenario, high accuracies are obtained for both frequency bands. The classification on the 6–10 GHz band showed an accuracy of 98.3%, while the performance of the algorithm on the 6–8 GHz band achieved a slightly higher accuracy of 99.6%, and the misclassifications are only obtained only in the false positive class. For multiclass classification, in the 6 to 8 GHz frequency band the performance is perfect with accuracy of 100%, and a high accuracy of 99.8% is observed in the 6 to 10 GHz band. The notable improvement in the performance accuracy of the SVM algorithm on the magnitude-only dataset can be attributed to the exclusion of the phase component from the measurements. In the near field, the phase of scattering parameters is highly sensitive to changes in distance. Small variations in the object’s position within the near-field region lead to a change in the angle of view of the jar with respect to the measurement system, which can result in significant phase variations. Given the movement of the jar, we anticipate a high phase fluctuations, that lead to an increase in the complexity of the dataset. On the other hand, the situation differs in the classifications involving water, where comparable and almost perfect accuracies were achieved with both dataset types. This can be attributed to the high contrast in permittivity between water and the contaminant materials, making the classification process simpler. Instead, the contrast in permittivity between oil and the contaminants is relatively lower than that between water and the same contaminants. This results in complexity in the classification process with oil jar samples and increases the errors in performance accuracy.

In the concluding part of the dataset construction section and referring to Fig. 13a, b, we proposed the idea of selecting a specific subrange within the frequency bandwidths to carry out the measurements. This selection is based on the favorable transmission characteristics observed in the amplitude plot of the scattering parameters (Fig. 13a, b). The effectiveness of this proposal is validated in the results, where the classifier algorithm exhibits improved performance when using only data from 6 to 8 GHz for oil-based products.

**Table 8:** Classification results on only amplitude dataset nature for oil.

Dataset split (training-test)	Type of classification	Frequency band [start : step : end] GHz	Confusion matrices (on test set)	Accuracy (%)
250–240 samples	Binary	[6 : 0.4 : 10]	$$\left[ \begin{array}{ccc} 76 &{} 4 \\ 0 &{} 160 \end{array}\right]$$	98.3
250–240 samples	Binary	[6 : 0.4 : 8]	$$\left[ \begin{array}{ccc} 78 &{} 2 \\ 0 &{} 160 \end{array}\right]$$	99.6
290–200 samples	Multiclass	[6 : 0.4 : 10]	$$\left[ \begin{array}{ccc} 100 &{} 0 &{} 0 \\ 0 &{} 49 &{} 1 \\ 0 &{} 0 &{} 50 \end{array}\right]$$	99.8
290–200 samples	Multiclass	[6 : 0.4 : 8]	$$\left[ \begin{array}{ccc} 100 &{} 0 &{} 0 \\ 0 &{} 50 &{} 0 \\ 0 &{} 0 &{} 50 \end{array}\right]$$	100

### Conclusion and perspectives

The imperative need to detect physical contaminants inside food and beverage products before market distribution prompted our focus on developing an MW sensing system to evaluate food products with varying ingredients. Considering the prevalence of water and oil as primary components in many food and beverage items, we introduced a versatile methodology applicable to both mediums. This study addressed the integration of MW sensing technology with machine learning tools for detecting contaminants in food and beverage products. The methodology encompassed a comprehensive examination of the MW sensing system, spanning the selection of suitable frequency bands to the system performance in the near-field radiated region. Our process began with the collection of scattering parameters to form datasets, subsequently proceeding to the classification stage employing the SVM learning algorithm. Notably high accuracy was achieved in both binary and multiclass classifications, encompassing datasets with complex numbers and those containing the amplitude part only.

While our study has made significant contributions to employing MW sensing technology enhanced by ML for the non-invasive evaluation of food products, it is essential to recognize its limitations to provide a comprehensive understanding of our findings. The main limitations are in the algorithm training phase. First of all, the numbers of samples used here for training were the minimum required to obtain a good classification performance. Then, in the multiclass analysis, we tried to include more contaminant materials, but, with the used SVM algorithm, the accuracy of the classification got worse. Hence, as part of our future work, we aim to enhance the robustness of our system by investigating other ML algorithms, incorporating additional contaminant materials, particularly in the context of multiclass classification. Moreover, we plan to extend our experimental analysis to a wider range of food and beverage products as well as conduct the system testing in industrial environments.
